# Chromoblastomycosis caused by *Fonsecaea pedrosoi*: a case report and literature review

**DOI:** 10.3389/fmed.2026.1842447

**Published:** 2026-05-28

**Authors:** Yuyun Wang, Lu Wang, Zhen Li, Yuanqing Qu, Yuan Liu

**Affiliations:** Department of Laboratory Medicine, The General Hospital of Western Theater Command of People’s Liberation Army (PLA), Chengdu, Sichuan, China

**Keywords:** chromoblastomycosis, combination therapy, *Fonsecaea pedrosoi*, itraconazole, ITS (internal transcribed spacer) region sequencing

## Abstract

*Fonsecaea pedrosoi*, a pathogenic fungus within the order Chaetomycosis of the family Dematiaceae, is the primary causative agent of Chromoblastomycosis (CBM). Its clinical manifestations are diverse and lack specificity, often being confused with other skin diseases, which can easily lead to misdiagnosis or missed diagnosis. This article reports a case of a 36-year-old immunocompromised female with chronic infection caused by *F. pedrosoi* following minor trauma to the right knee. The patient presented with plaque-like hyperplasia, pruritus, desquamation, and suppuration of the local skin over the right knee, with persistent and refractory clinical course. The definite diagnosis was ultimately established through mycological, histopathological, and molecular biological examinations, followed by appropriate therapeutic intervention. This case indicates that chromoblastomycosis is prone to missed or delayed diagnosis due to its atypical clinical manifestations. Moreover, immunocompromised patients are more likely to experience prolonged disease course and slow recovery, highlighting the critical importance of accurate diagnosis and rational selection of treatment regimens. Combined with a literature review, it analyzes the clinical characteristics and key points in diagnosis and treatment of the disease, explores approaches to strengthen the awareness of clinical monitoring, and aims to improve the overall public health awareness of the disease.

## 1 Introduction

Chromoblastomycosis (CBM), also known as chromomycosis, is a chronic granulomatous disease affecting the skin and subcutaneous tissue, primarily caused by dematiaceous fungi (1). Common pathogens responsible for human infections include *Fonsecaea, Cladophialophora, Rhinocladiella*, *Phialophora*, *Exophiala*, *Alternaria*, *Chaetomium*, etc., with *Fonsecaea* and *Cladophialophora* being the predominant ones (2, 3). The disease was first reported by Pedroso in 1920 (4). Since then, it has been reported worldwide, but exhibits a distinct geographical distribution, being more common in tropical and subtropical regions such as Latin America, Africa, and Asia (5, 6). In northern China, infections caused by *C. carrionii* are more prevalent, while in southern China, infections due to *F. pedrosoi* are more common (7). The clinical manifestations of CBM are non-specific, and it is often misdiagnosed as skin tuberculosis, eczema, other superficial fungal infections, or other dermatological conditions such as skin tumors (8–10). This article reports the diagnostic and therapeutic process of a patient with *F. pedrosoi* infection.

## 2 Case presentation

A 36-year-old female patient was admitted to the dermatology clinic of our hospital on July 2, 2024, due to “partial skin damage with plaque-like hyperplasia on the right knee for more than 2 years.” The patient had systemic lupus erythematosus (SLE) for more than 10 years, accompanied by lupus nephritis and nephrotic syndrome, and was undergoing regular treatment and follow-up.

According to the patient’s account, more than 2 years prior, the right knee was bruised by a tree trunk during work, with associated skin damage and a small amount of bleeding. Simple disinfection was performed, but no further attention was paid. After the skin lesions scabbed, occasional itching occurred. However, the lesion area gradually enlarged and thickened, with dark red plaques appearing, accompanied by obvious itching. Ulceration occurred after scratching, often with pale yellow or purulent exudate. The patient had visited multiple hospitals, but the effect of topical drug treatment was poor (specific treatment regimen unknown).

**Figure 1:**
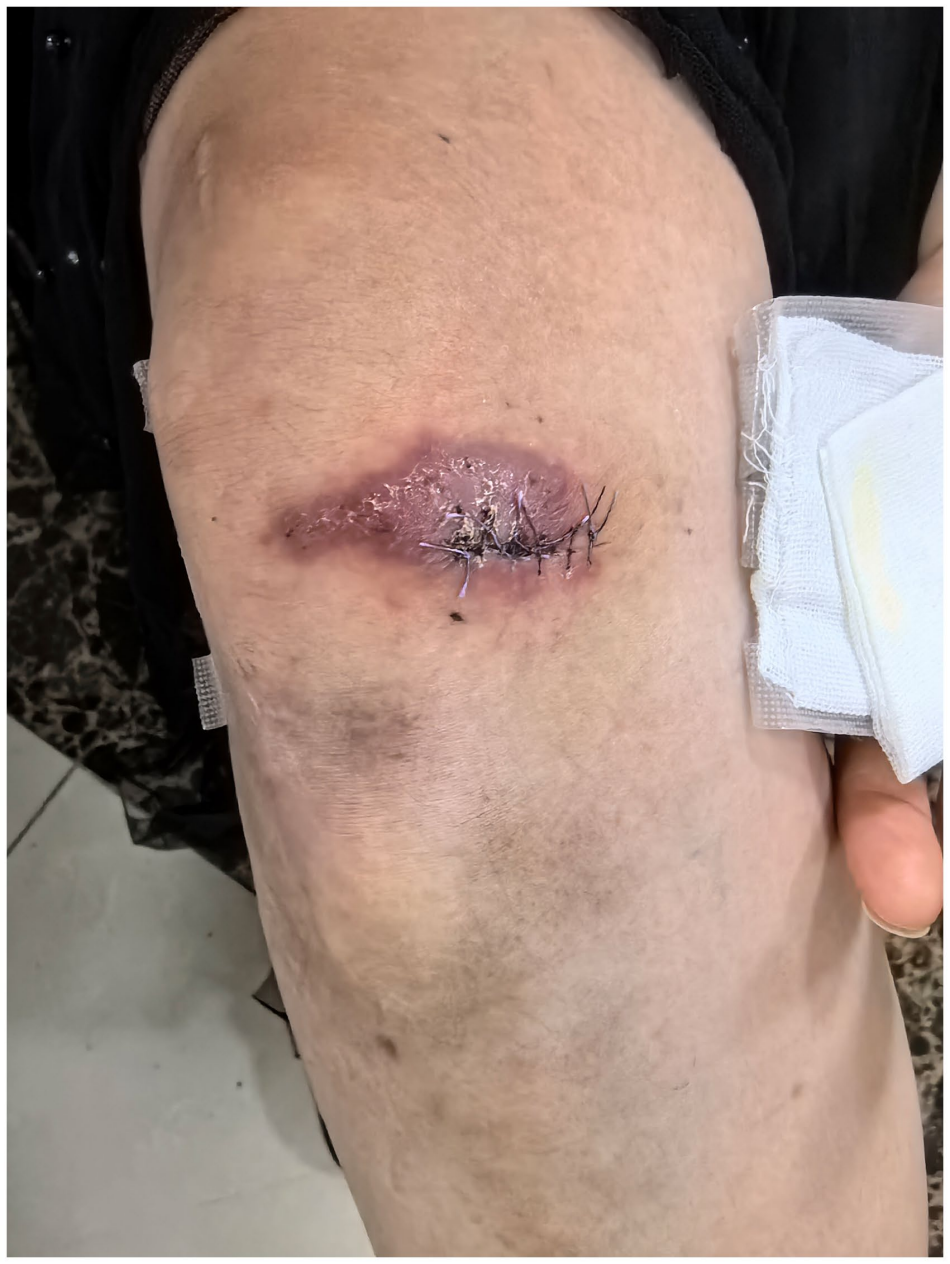
Clinical manifestation of chromoblastomycosis on the right knee of a 36-year-old immunocompromised female patient before antifungal treatment. A reddish-brown scar was observed on the right knee, with a surgical incision at the scar margin; the skin on the tibial aspect of the right lower leg appeared blackish-brown.

Physical examination revealed a reddish-brown scar measuring approximately 6 × 3 cm on the right knee with irregular margins, partially covered by crusts and white scales. A surgical incision of about 3 cm in length was noted at the edge of the scar, which was made for tissue specimen collection. The skin on the tibial side of the right lower leg presented with a blackish-brown discoloration (Figure 1). The preliminary diagnosis considered tinea, Majocchi granuloma, or cutaneous fungal granuloma. Clinicians collected skin scales from the lesion using the tape method. The adhered samples were stained with the fungal fluorescence reagent (FungusClear LIMING BIO) for 30–60 s and examined under a fluorescence microscope (OLYMPUS CX43) at 40 × magnification. No fungal spores or hyphae were observed, and the result was negative. Empiric antifungal treatment for skin infection was administered, but the effect was unclear.

**Figure 2:**
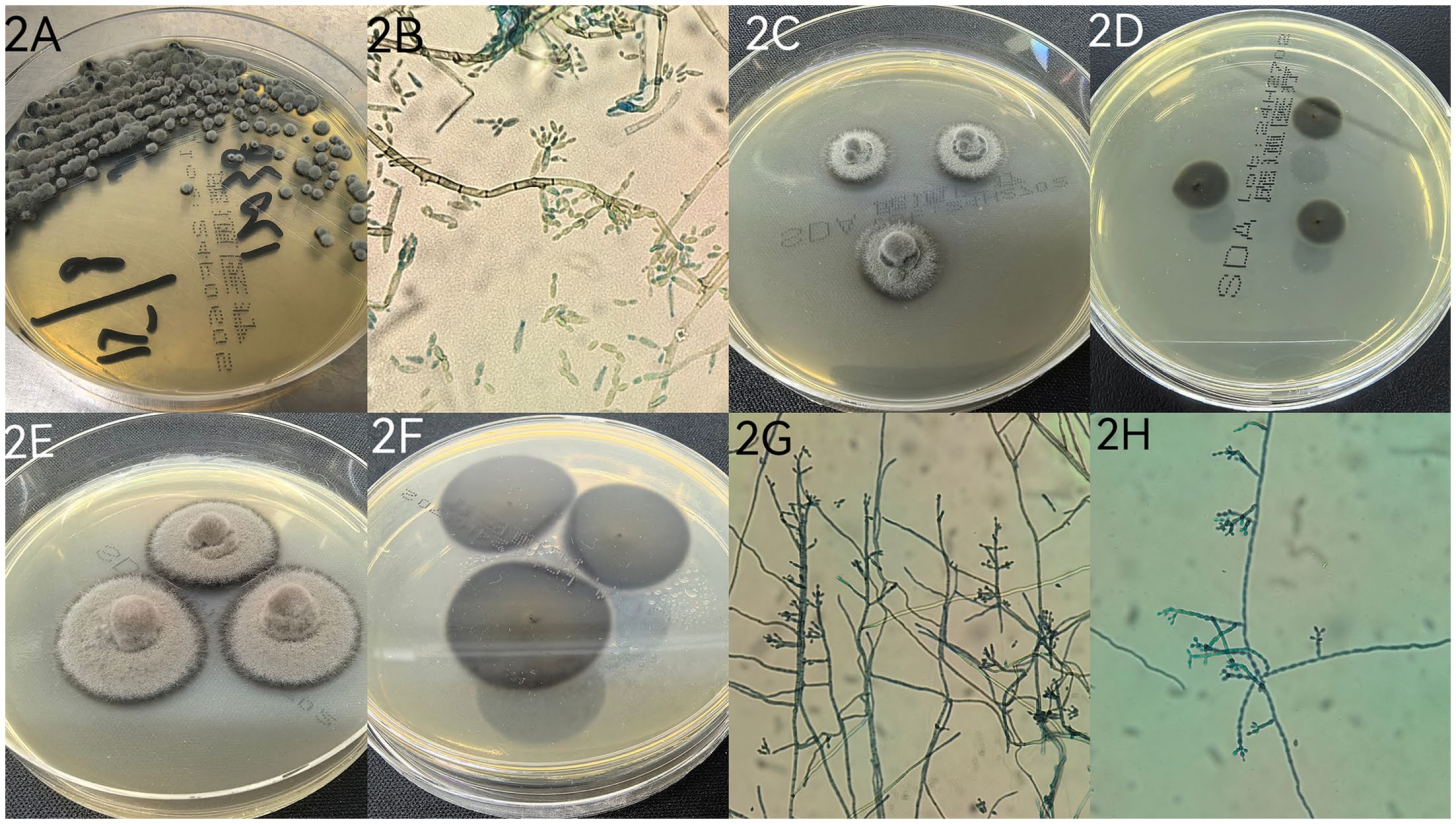
Morphological characterization of *Fonsecaea pedrosoi* through fungal culture and microscopic examination, illustrating colonial morphology at various incubation stages and highlighting distinctive ramoconidial sporulation structures. **(A)** Cultured on SDA at 35 °C for 7 days, the colony appears grayish-black with a surface of fine, grayish-white mycelial tufts. **(B)** Lactophenol cotton blue staining, observed under oil immersion lens (1000×), revealed thick, brown, branched and septate hyphae, and brown subglobose conidia, with ramoconidia formed at the branch ends. **(C,D)** After isolation and culture on SDA at 28 °C for 7 days, the colony was gray-white, round, centrally raised, velvety on the surface, and black on the reverse. **(E,F)** After isolation and culture on SDA at 28 °C for 15 days, the colony turned brownish-gray, round, with a black margin, central umbonate, velvety on the surface, and black on the reverse. **(G,H)** After microculture at 28 °C for 7 days and lactophenol cotton blue staining, observed under high-power microscope (400×), the ramoconidial structure was visible, with conidia arranged sympodially, and the conidia were round or ellipsoidal.

**Figure 3:**
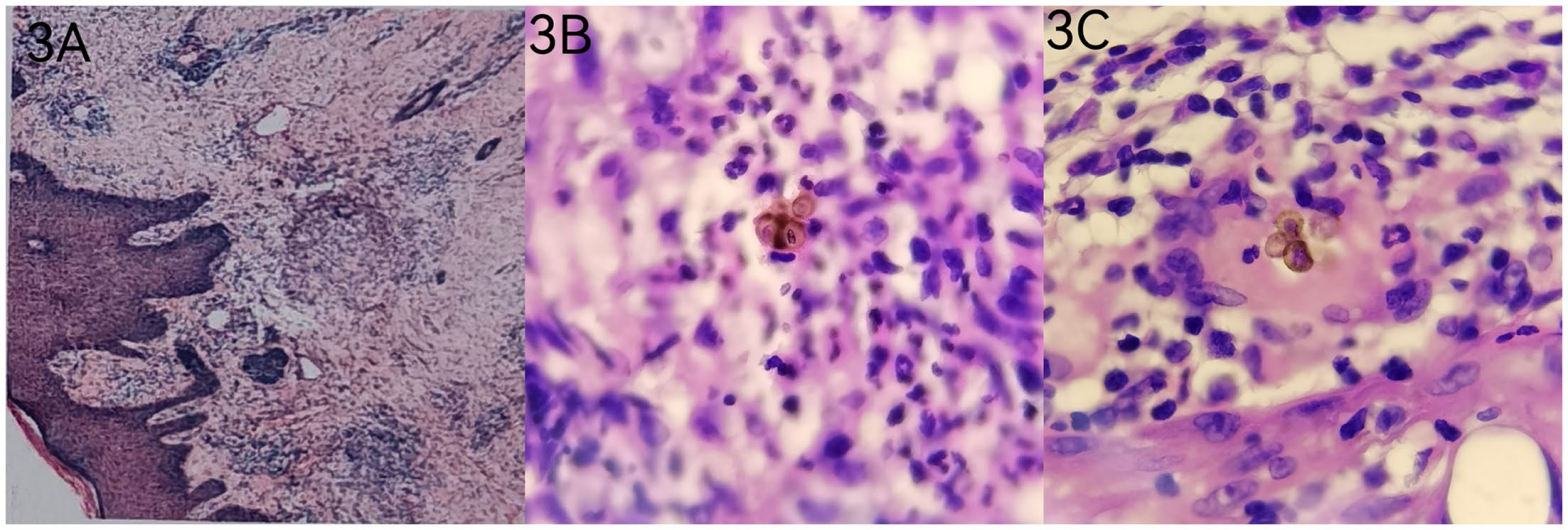
The histopathological examination of biopsy tissue from the right knee lesion reveals characteristic granulomatous inflammation and PAS-positive septate sclerotic bodies, confirming chromoblastomycosis. **(A)** The patient’s right knee joint shows hyperkeratosis with parakeratosis of the squamous epithelium, hyperplasia of the spinous layer, focal infiltration of neutrophils in the subepidermal region, and granulomatous nodule formation. **(B,C)** Observed under oil immersion lens (1000×), PAS staining shows clusters of brown, round, thick-walled septate spores.

On August 12, 2024, during follow-up, lesion tissue was collected for pathogen culture and pathological examination. Tissue fragments were inoculated onto Sabouraud Dextrose Agar (SDA), Columbia Blood Agar (BA), and chocolate agar, then incubated at 35 °C. After 3 days of incubation, a layer of small, grayish-black, fluffy colonies grew on SDA (Figure 2A). Membranous, light gray colonies also appeared on BA and chocolate agar, with indistinct morphology and color. The colonies on SDA were sampled by the tape method and subjected to lactophenol cotton blue staining. Under the oil immersion lens, brown, branched and septate hyphae as well as brown subglobose spores were observed (Figure 2B). Subsequently, further subculture was performed: the isolates were spot-inoculated onto SDA. The agar block method was used for fungal microcultivation. Potato dextrose agar (PDA) was prepared in a 90-mm culture dish and cut into four 5 × 5 mm blocks to serve as the culture substrate. A small amount of mycelium from the test strain was collected with an inoculation needle and spot-inoculated onto the peripheral side wall of each agar block, which was then covered with a coverslip. The plate was sealed, placed in a moist culture box, and incubated at 28 °C for subsequent morphological observation. After another 5 days of incubation, the colonies appeared grayish-white with black margins, round in shape, centrally convex, fluffy on the surface and black on the reverse side (Figures 2C,D). Lactophenol cotton blue staining was applied to the colonies from the microculture, and under the oil immersion lens, the sympodial arrangement of conidia and rhinocladiella-type sporulation were further observed (Figures 2G,H). With prolonged incubation, the colonies turned brownish (Figures 2E,F). Fungal culture (Figure 2) preliminarily suggested a genus within *Dematiaceae*. On August 19, 2024, the pathological diagnosis report indicated hyperkeratosis with parakeratosis of the squamous epithelium, acanthosis, focal infiltration of neutrophils beneath the epidermis, and granulomatous nodule formation in the right knee joint (Figure 3A). Based on the results of fungal culture and lactophenol cotton blue staining, the histopathological sections were re-stained and observed. Eventually, in the histopathological sections processed with periodic acid-Schiff (PAS) staining, brown, round and septate chlamydospores were observed under the oil immersion lens (Figures 3B,C).

At this point, the pathogenic fungus was identified. To further confirm the result, the cultured colonies were sent to Jingming Haorui, a third-party laboratory, for internal transcribed spacer (ITS) region sequencing. Finally, ITS sequencing and NCBI BLAST alignment^1^ showed 96.61% sequence identity and 99% query coverage with *Fonsecaea pedrosoi* (NR_130652.1). Meanwhile, *in vitro* antifungal susceptibility testing was performed to determine the minimum inhibitory concentration (MIC) in accordance with the guidelines of Clinical and Laboratory Standards Institute (CLSI) document M38-A2. We used a commercial 9-drug antifungal susceptibility plate (Thermo Fisher Scientific) to assess filamentous fungal susceptibility. Fungal colonies from the test strain were collected with a cotton swab and suspended in sterile physiological saline containing Tween to yield a homogeneous suspension adjusted to 0.5 McFarland turbidity. Then 100 μL of this suspension was added to 11 mL of YeastOne inoculation broth and mixed thoroughly. Next, 100 μL of the resulting broth was dispensed into each well of the antifungal susceptibility plate. The inoculation was completed within 15 min. After sealing the wells with adhesive film, plates were incubated at 35 °C in a non-CO_2_ incubator for 48 h. Finally, results were read. The results of *in vitro* antifungal susceptibility testing were as follows: Amphotericin B MIC 1 μg/mL; 5-fluorocytosine MIC 2 μg/mL; Fluconazole MIC 32 μg/mL; Voriconazole MIC 0.12 μg/mL; Itraconazole MIC 0.25 μg/mL; Posaconazole MIC 0.12 μg/mL; Anidulafungin MIC 8 μg/mL; Micafungin MIC 8 μg/mL; Caspofungin MIC 1 μg/mL.

**Figure 4:**
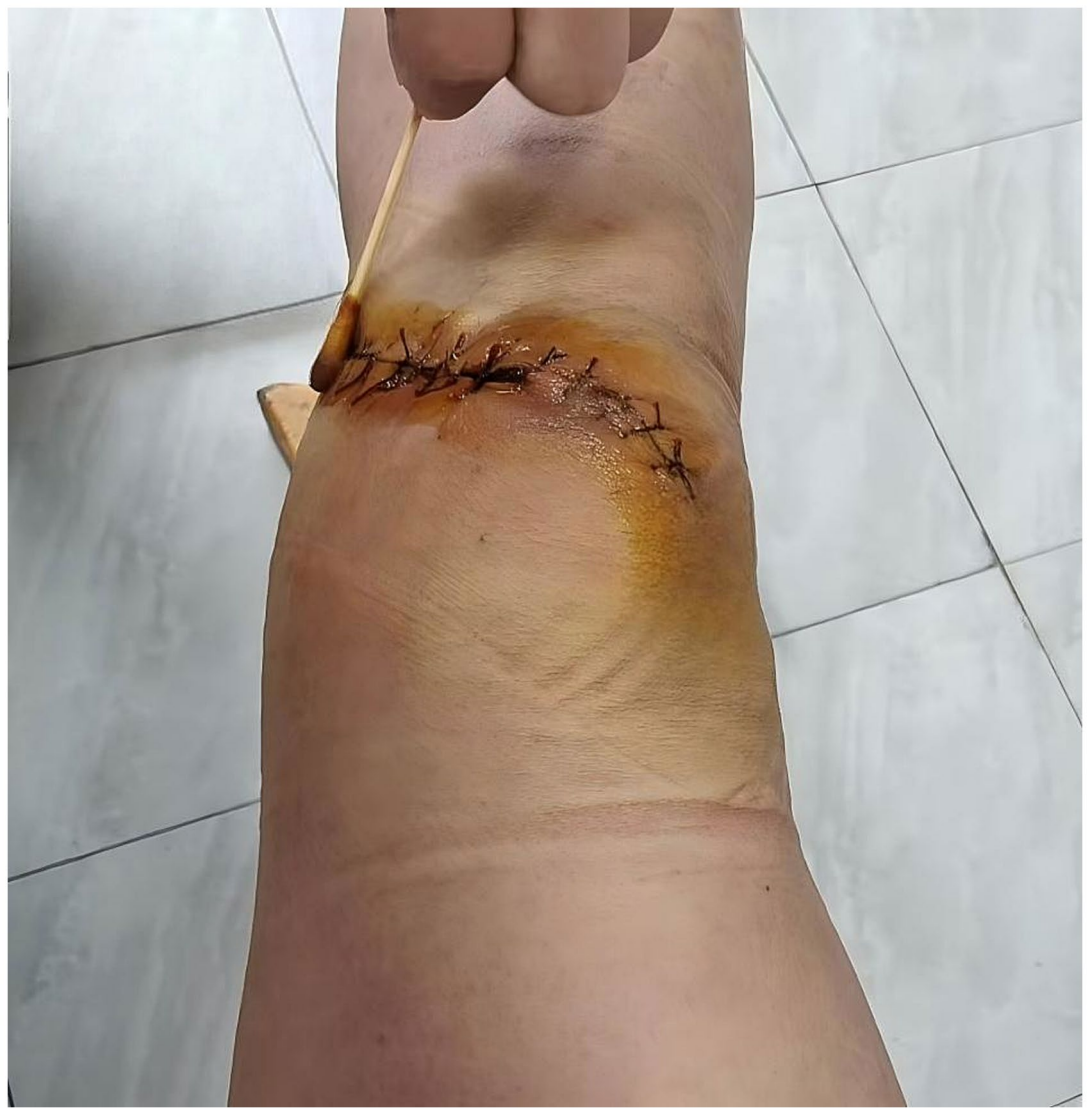
Postoperative appearance of the lesion in the right knee after surgical debridement and excision of the affected tissue for chromoblastomycosis.

Based on the results of pathogen identification and antifungal susceptibility testing, a treatment regimen was formulated by the physicians, consisting of oral administration of itraconazole at a daily dose of 200 mg. Due to the patient’s impaired immune function and long-term use of corticosteroids, the recovery of skin lesions was slow. After thorough review of relevant literature and discussions with clinicians, combined with the patient’s condition, hyperthermia therapy was added to the treatment. The patient was advised to strengthen nutrition to improve immune function. Three months later, the patient’s skin lesions showed slight improvement. However, after 6 months of regular treatment and follow-up visits, there was still no significant improvement in the skin lesions. Having suffered from underlying diseases for many years, the patient could not psychologically accept the unfortunate fact that a wound caused by a minor collision had developed into chronic chromoblastomycosis. In addition, due to long-term oral administration of antifungal drugs, the patient was worried about potential additional damage to the body, and gradually stopped follow-up visits, resulting in loss to follow-up. Later, we learned that the patient opted for local surgical treatment in August 2025 to resect the lesional tissue (Figure 4). The patient’s diagnosis and treatment process is detailed in the Supplementary materials.

## 3 Literature review

To further explore the epidemiological characteristics and clinical manifestations of the disease, a comprehensive search was conducted across China National Knowledge Infrastructure (CNKI), Wanfang Database, and PubMed using search terms such as “*Fonsecaea pedrosoi*,”“*F. pedrosoi*” and “chromoblastomycosis” from 1999 to 2024. A total of 82 articles encompassing 98 cases were retrieved and analyzed, including 32 cases in China (with the current case being a new addition) and 66 international cases.

### 3.1 General characteristics

*Fonsecaea pedrosoi* infection can affect both immunocompetent and immunocompromised individuals. In China, the male-to-female ratio was 3.6:1, with a mean age of onset of approximately 52.5 years. Internationally, the male-to-female ratio was 2.7:1, with a mean age of onset of around 50 years. Globally, 62 cases were reported in Asia (including 32 in China), predominantly in southern provinces. Many patients had identifiable risk factors such as a history of trauma or bites.

### 3.2 Clinical features

Infections caused by *F. pedrosoi* are mainly cutaneous diseases. All 32 domestic cases presented as cutaneous involvement. Among 66 foreign cases, 53 (80.3%) presented as cutaneous involvement, with another 13 cases manifesting as other types of infections, including 5 cases of central nervous system infection (brain abscess) (7.6%) (11, 12), 3 cases of ocular infection (4.5%), 2 cases of joint and bone infection (3.0%), 1 case of skull base and middle ear cavity infection (1.5%) (13), 1 case of mediastinal infection (1.5%) (14), and 1 case of nasal and paranasal sinus infection (1.5%) (15). Lesions were most commonly located on the limbs, face, and neck, with rare occurrences on the buttocks, chest, and abdomen (Table 1). Clinical manifestations were diverse but exhibited distinct stage-specific characteristics (Table 2).

**Table 1:** Distribution of anatomical sites of skin lesions in 85 reported cases of chromoblastomycosis caused by *Fonsecaea pedrosoi*.

Skin lesions site	Cases	Proportion (%)
Facial and neck regions	6	7.1
Upper limbs	27	31.8
Thoracic and abdominal regions	2	2.4
Buttocks	4	4.7
Lower limbs	46	54.1

**Table 2:** Stage-specific clinical manifestations of cutaneous chromoblastomycosis caused by *Fonsecaea pedrosoi* in 85 reported cases, classified by disease progression stage (early, progressive, and advanced).

Features of skin lesions	Early stage (cases)	Progressive stage (cases)	Advanced stage (cases)
Erythema	38	25	15
Papules	30	21	10
Pustules	7	9	7
Nodules	25	29	18
Scales	19	26	19
Plaques	22	40	33
Verrucous hyperplasia	14	40	33
Ulcers	8	18	27
Sinus openings	4	7	14
Crusts	17	23	20
Scar-like lesions	6	11	28
Black dots	12	14	11
Induration	11	13	12
Joint dysfunction	4	8	15

The same patient may present with multiple manifestations of skin lesions in the early, progressive, and advanced stages.

### 3.3 Diagnosis and treatment

Diagnosis of chromoblastomycosis primarily relies on mycological and histopathological examinations, with molecular techniques (e.g., ITS region sequencing) enabling specific pathogen identification.

Treatment modalities include drug therapy, surgery, physical therapy, and combination therapy (16). Itraconazole was the preferred initial drug (66.7%), with Terbinafine commonly used as an adjunct (48.1%). Surgical excision (17.3%), sometimes requiring skin grafting for larger lesions, was used for localized cutaneous lesions. Physical therapies (27.2%)—including thermotherapy, cryotherapy, and laser therapy—were often combined with medical interventions, showing substantial efficacy.

Post-treatment outcomes: 36 cases (44.44%) achieved recovery, 39 cases (48.15%) showed improvement, and 6 cases (7.41%) improved initially but relapsed after discontinuing treatment, resulting in an overall effectiveness rate of 92.59% (Table 3).

**Table 3:** Treatment modalities and clinical outcomes in 81 evaluable cases of chromoblastomycosis caused by *Fonsecaea pedrosoi* (literature review, 1999–2024): antifungal pharmacotherapy, surgical excision, physical therapies, and post-treatment response rates.

Treatment regimens	Cases	Cured cases	Improved cases	Uncured cases
Pharmac-otherapy	Azoles	Itraconazole	11	4	6	1
		Itraconazole + Physical therapy	8	3	5	
		Itraconazole + Surgical treatment	3	2	1	
		Itraconazole + Potassium iodide	2		1	1	Azoles	Itraconazole + Ebastine	1		1	
	Itraconazole + Liuwei Lotion poultice	1		1			
	Voriconazole	3			3		
	Fluconazole	2		2			
	Pharmac-otherapy	Allylamines	Terbinafine	7	6	1	
Terbinafine + Physical therapy			5	3	2	
Terbinafine + Surgical treatment			3	2	1		Azoles + Allylamines	Itraconazole + Terbinafine	7	3	4	
Itraconazole + Terbinafine + Physical therapy		9		8	1		
Itraconazole + Terbinafine + Surgical treatment		3	2	1			
Itraconazole + Terbinafine + Other drugs		2	1	1			
Polyenes		Amphotericin B + Itraconazole	2	1	1	
		Amphotericin B + Itraconazole + Surgical treatment	1		1	
Immunomo-dulators		Imiquimod	3	3		
		Imiquimod + Itraconazole	1	1		
		Imiquimod + Itraconazole + Terbinafine	3	1	2	
Surgical- treatment			Surgical resection	4	4		
Total			81	36	39	6

2 cases refused treatment, 1 case had an unknown prognosis, and 1 case abandoned treatment; these were not included in the statistics.

## 4 Discussion

*Fonsecaea pedrosoi*, a pathogenic fungus in the order *Chaetomycetales* and family *Herpotrichiellaceae*, is the main cause of chromoblastomycosis (CBM) (17). This disease primarily affects people in tropical and subtropical areas who have occupational exposure to soil and plant material (5, 18, 19).

*F. pedrosoi* induced CBM exhibits a wide range of clinical manifestations with low specificity, contributing to a high misdiagnosis rate (10). In the initial cutaneous stage, erythema (44.7%) and small papules (35.3%) are common. As the disease advances, patients develop nodules, plaques, verrucous hyperplasia, crusts, and scales, often with mild discomfort and local lymphadenopathy. In severe cases, ulcers and sinus tracts may form, complicating treatment and resulting in scarring after resolution. Prolonged disease duration can affect joints (17.6%), causing dysfunction and, in severe cases, bone destruction or progression to squamous cell carcinoma (10, 20, 21).

Diagnosis of *F. pedrosoi* CBM primarily relies on mycological and histopathological examinations. Direct microscopic examination and fungal culture are crucial diagnostic tools, with the latter serving as the gold standard (22). Molecular analyses (e.g., ITS region sequencing) can further refine pathogen identification. Histopathologically, the presence of characteristic brown, septate chlamydospores (sclerotic bodies) is key diagnostic evidence (21). In this case, combining histopathological examination with ITS sequencing allowed for the precise identification of *F. pedrosoi*, enabling the early initiation of suitable antifungal therapy.

For immunocompromised patients, like the one in this case with systemic lupus erythematosus (SLE) undergoing long-term corticosteroid therapy, preventing fungal infections is particularly crucial. These patients often have compromised skin barrier function and weakened immune defenses, making it essential to enhance daily protective measures to avoid direct skin contact with environmental sources that may contain pathogenic fungi. Treating fungal infections in such cases requires extended treatment courses to minimize the risk of recurrence. In this instance, the patient underwent a 9-month course of itraconazole therapy, supplemented by surgical debridement, which led to significant clinical improvement.

The laboratory testing process for *F. pedrosoi* is crucial for early infection control. A comprehensive diagnostic approach, which includes histopathological examination, fungal culture, and molecular biological identification, is essential. While fungal culture requires more time, it is fundamental for subsequent strain identification and drug susceptibility testing. ITS region sequencing,on the other hand, can quickly and accurately identify the pathogenic fungus, providing a basis for early targeted treatment. Timely and precise laboratory testing enables a clear diagnosis, effectively controlling infection spread and limiting the expansion of skin lesions.

## 5 Conclusion

Limited laboratory resources and inadequate awareness of rare fungal infections among clinicians render primary hospitals prone to misdiagnosis of *F. pedrosoi* infection. Strengthening professional training can improve clinicians’diagnostic proficiency. For suspected cases unconfirmed by routine tests, specimens should be timely transferred to superior hospitals or qualified laboratories to avoid irrational drug use. Establishing regional referral and consultation mechanisms facilitates resource sharing and technical cooperation, elevating the overall diagnosis and treatment level of rare fungal infections. Furthermore, clinical humanistic care deserves attention. Although such cases are rare for clinicians, they often cause long-term physical and psychological distress to patients. Therefore, medical staff should provide adequate care and empathy to help patients recover smoothly.

This case report has certain limitations. As it is only a single-case study, the findings may not be applicable to all patients with *F. pedrosoi* CBM. Further research with larger sample sizes and comprehensive microbiological profiling is necessary to develop more robust diagnostic and therapeutic guidelines for this rare fungal infection.

## Data Availability

The original contributions presented in the study are included in the article/Supplementary material, further inquiries can be directed to the corresponding authors.

## Footnotes

1
http://blast.ncbi.nlm.nih.gov/
