# Supplementary figures and images for: Chromoblastomycosis caused by Fonsecaea pedrosoi: a case report and literature review
Source: Front Med (Lausanne). 2026 May 28;13:1842447. doi: 10.3389/fmed.2026.1842447 (PMC13262189; doi:10.3389/fmed.2026.1842447)

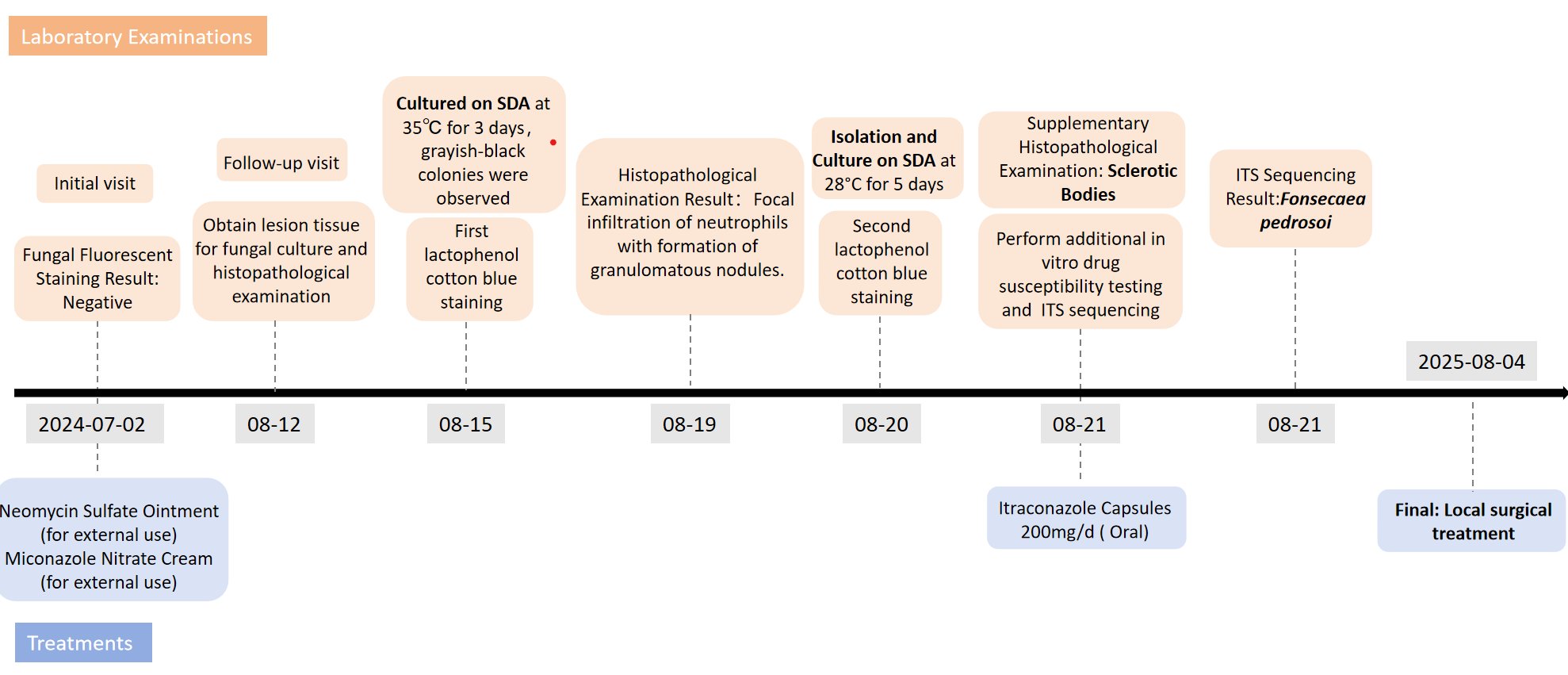

Supplement: Supplementary file 1 [file Image_1.png]
